# Evaluating the nutritional content of an insect-fortified food for the child complementary diet in Ghana

**DOI:** 10.1186/s40795-020-0331-6

**Published:** 2020-04-02

**Authors:** Megan E. Parker, Stephanie Zobrist, Herman E. Lutterodt, Cyril R. Asiedu, Chantal Donahue, Connor Edick, Kimberly Mansen, Gretel Pelto, Peiman Milani, Shobhita Soor, Amos Laar, Cyril M. Engmann

**Affiliations:** 1 PATH, Maternal, Newborn, and Child Health and Nutrition, 2201 Westlake Ave, Suite 200, Seattle, WA 98121 USA; 2 Department of Food Science and Technology, Kwame Nkrumah University of Science and Technology, Kumasi, Ghana; 3 Division of Nutritional Sciences, Cornell University, Ithaca, New York, USA; 4 Sight and Life Foundation, Kaiseraugst, Switzerland; 5 Aspire Food Group, Kumasi, Ghana; 6 Department of Population, Family and Reproductive Health, School of Public Health, University of Ghana, Accra, Ghana; 7 Department of Global Health, University of Washington, Seattle, Washington, USA; 8 Department of Pediatrics, University of Washington & Seattle Children’s Hospital, Seattle, Washington, USA

**Keywords:** Edible insects, Animal-source food, Complementary food, Nutrient profile, Ghana

## Abstract

**Background:**

Due to rising food insecurity, natural resource scarcity, population growth, and the cost of and demand for animal proteins, insects as food have emerged as a relevant topic. This study examines the nutrient content of the palm weevil larva (*Rhynchophorus phoenicis*), a traditionally consumed edible insect called *akokono *in Ghana, and assesses its potential as an animal-source, complementary food.

**Methods:**

*Akokono* in two “unmixed” forms (raw, roasted) and one “mixed” form (*akokono*-groundnut paste) were evaluated for their macronutrient, micronutrient, amino acid, and fatty acid profiles.

**Results:**

Nutrient analyses revealed that a 32 g (2 tbsp.) serving of *akokono*-groundnut paste, compared to recommended daily allowances or adequate intakes (infant 7–12 months; child 1–3 years), is a rich source of protein (99%; 84%), minerals [copper (102%; 66%), magnesium (54%; 51%), zinc (37%; 37%)], B-vitamins [niacin (63%; 42%), riboflavin (26%; 20%), folate (40%; 21%)], Vitamin E (a-tocopherol) (440%; 366%), and linoleic acid (165%; 108%). Feed experiments indicated that substituting palm pith, the typical larval diet, for pito mash, a local beer production by-product, increased the carbohydrate, potassium, calcium, sodium, and zinc content of raw *akokono*. *Akokono*-groundnut paste meets (within 10%) or exceeds the levels of essential amino acids specified by the Institute of Medicine criteria for animal-source foods, except for lysine.

**Conclusions:**

Pairing *akokono* with other local foods (e.g., potatoes, soybeans) can enhance its lysine content and create a more complete dietary amino acid profile. The promotion of *akokono* as a complementary food could play an important role in nutrition interventions targeting children in Ghana.

## Background

Maternal and child undernutrition remains pervasive in low- and middle-income countries (LMICs) and together constitutes the leading underlying cause of child morbidity and mortality [1]. Against a backdrop of global trends such as urbanization, growing populations, and rising incomes, the global food system faces the looming challenge of meeting the world’s evolving nutritional needs [2]. Animal-source foods (ASFs) are important components of diverse diets, providing protein and essential micronutrients that promote growth and development. Increasing evidence points to the positive impact of animal-source proteins on linear growth and physical and cognitive development in children [3–7]. The World Health Organization recommends that children 6 to 23 months of age consume ASFs daily [8]. Yet, ASFs are often costly and remain out of reach for many low-income households [5]. The global demand for ASFs in many LMICs is expected to rise substantially over the coming decades [9]. It is projected that current livestock production practices will be unable to sustainably meet this growing demand [10, 11]. Thus, edible insects are emerging as a potential avenue to sustainably improve nutrition.

### Insects as food

Entomophagy, the consumption of insects as food, is a long-standing practice in many cultures around the world and has played a role in the history of human nutrition. The Food and Agriculture Organization estimates that approximately 2 billion people worldwide consume insects as part of their diets, [12] and Jongema [13] has documented over 2000 species of edible insects consumed globally. In comparison to traditional livestock, edible insect production has the potential to positively contribute to environmental sustainability due to lower resource requirements, feed-conversion rates, and greenhouse gas emissions [14, 15].

In the context of locally-sourced diets in LMICs, where the burden of malnutrition is highest, edible insects can contribute essential nutrients necessary to augment dietary quality and diversity among individuals who primarily consume cereal-based foods [16]. However, the nutritional profiles of edible insects indicate substantial variability both between and within species [17, 18] and comprehensive analyses of both macronutrient and micronutrient content are lacking [19].

### The Ghanaian context

Malnutrition remains a pressing concern in Ghana, where one-fifth (19%) of children under five suffer from chronic malnutrition (stunting), 5% suffer from acute malnutrition (wasting), and two-thirds (66%) of children between 6 and 59 months of age are anemic [20]. The typical complementary diet of Ghanaian children 6 to 23 months is composed of grains, fruits and vegetables, roots and tubers, and some animal products; only 13% of children of this age met the criteria for a minimally acceptable diet [20].

In Ghana, edible insects are already included in certain traditional diets. In a countrywide survey of 2000 individuals, almost one-third reported consuming edible insects, the most common being the palm weevil larva (*Rhynchophoris phoenicis*) [21]. Palm weevil larvae, known locally as *akokono* in Ghana, are consumed across regions of Africa, Asia, and Latin America [12]. In Ghana, the *akokono* typically feed on the pith of palm trees that are felled for harvesting palm wine and, when farmed, can be available year round [22, 23]. When harvested for food, *akokono* is typically fried, roasted as a kebab, or boiled in a soup (personal communication with Aspire Food Group, June 2017). According to a recent study, *akokono* was largely perceived to be an acceptable complementary food source by caregivers in the Brong-Ahafo Region of Ghana [22].

Previous studies investigating the nutritional composition of palm weevil larvae have typically reported high protein, fat, and mineral content, but there is variability between studies [16, 24–31]. This variability may be explained by a number of factors, including differences in insect habitat, geographic location, developmental stage, and feed composition, as well as the study measurement methods [17]. To our knowledge, no studies to date have examined the nutritional profile of the palm weevil larva in Ghana.

### Purpose

We hypothesize that the consumption of *akokono* could help infants and young children meet essential amino acid [32] and micronutrient requirements and offset the need for daily consumption of traditional ASFs. The purpose of this study is to examine the nutrient profile of *akokono* (raw, roasted) to characterize the potential of this insect as an ingredient in complementary foods. We aimed to evaluate *akokono* and *akokono*-fortified foods as alternatives to conventional ASFs and examine the composition of their nutrient profiles based on feed type.

## Key messages

- *Akokono* is a good source of several essential nutrients including protein, fat, zinc, and B-vitamins that are important for child growth and development.
- Combining *akokono* with local groundnuts can improve the amino acid and vitamin profiles of the nut-based food.
- Substituting the traditional feed substrate of palm pith with pito mash improves both the macronutrient and micronutrient profile of *akokono.*
- Optimizing the nutrient content of this insect via manipulation of feed substrate is an area in need of further research.
- *Akokono* has the potential to improve child health and nutrition outcomes and can be incorporated into nutrition interventions.

## Methods

We examined the nutrient profile of *akokono* in two “unmixed” forms: raw and roasted. Additionally, three “mixed” *akokono* products were developed at the Kwame Nkrumah University of Science and Technology (KNUST): *akokono* flour, *akokono*-yam biscuit, and *akokono*-groundnut paste. The *akokono*-groundnut paste was chosen by the lead food technologist as the most promising food product for further testing and development as a potential child complementary food. Macronutrient composition, amino acid profile, fatty acid profile, mineral, and vitamin tests were conducted on these three forms of *akokono* as described below. Additionally, we measured the macronutrient and mineral composition of raw *akokono* fed two different types of feed materials, pito mash and palm pith, as well as that of the feed materials themselves.

Macronutrient composition, amino acid profile, fatty acid profile, and mineral tests (except for iron) were conducted at KNUST in Ghana (described below). All tests conducted at KNUST represent the mean of duplicate analysis. Eurofins Scientific performed all the vitamin tests and examined the iron content of raw akokono, roasted *akokono, and akokono*-groundnut paste; the Eurofins results represent the mean of triplicate analysis (described below). KNUST used identical macronutrient and mineral testing procedures on the *akokono* and the feed materials (pito mash, palm pith).

### Preparation

The *akokono* were bred in a warehouse in the Ashanti Region, fed with palm pith and sugar water, and procured from the Aspire Food Group facility in Kumasi. At the time of testing, *akokono* were between 28 and 35 days old (same development stage). Roasted samples were prepared by first pan-frying the *akokono* for 5 min followed by dry roasting at low heat for 15 min. Using a recipe created at the KNUST food technology laboratory, the *akokono*-groundnut paste was produced by mixing and then milling dry-roasted *akokono* (30%) together with local groundnuts (70%) and adding a small amount of canola oil (2 mL oil per 100 g paste) to improve the organoleptic properties of the final product. Finally, KNUST tested the *akokono-*groundnut paste to assess shelf-life stability, microbial safety, and chemical deterioration of the product at 0, 7, and 14 days as required by the Ghana Food and Drugs Authority (FDA). The safety data satisfied the Ghana Standards Authority (GSA) requirements for similar groundnut-based products (data not presented herein) [33]. To test the feed materials, palm pith was harvested from purchased felled palm trees in the Ashanti Region, and the pito mash, a by-product of local pito (beer) brewing, was purchased from a local market.

### Proximate (macronutrient) analysis

AOAC standard methods [34] were used to determine the proximate composition (moisture, ash, crude fat, protein, and fiber) of the *akokono* samples in duplicate. Significant differences between the macronutrient contents of the three forms of *akokono* were analyzed using one-way ANOVAs.

### Amino acid profile

The amino acid content was determined by first digesting the sample for 24 h at 110 °C using 37% HCl. Pre-column derivatization was done using *o*-phthalaldehyde 3-mercaptopropionic acid and 9-fluroenylmethyl chloroformate according to the method described by Schwarz, Roberts, & Pasquali [35]. An aliquot of 100 μL was injected into a Shimadzu high-performance liquid chromatography (HPLC) fitted with a Shimadzu 10AxL Fluorescence detector.

Mobile phase A was composed of 40 mM CH_3_COONa at pH 7.8 and mobile phase B composed of CH_3_CN: CH_3_OH: H_2_O (45:45:10 v/v/v) at a flow rate of 1 ml/min, passed through a Phenomenex column (3.5 μm, 4.6 mm ID, 15 cm) at 40 °C with run time of 60 min at the following wavelengths: Exitation: 340 nm; Emission: 450 nm.

A gradient elution program was used as described herein: 0 to 10 min (20% mobile phase B), 10 to 20 min (50% mobile phase B), 20 to 30 min (60% mobile phase B), 30 to 35 min (80% mobile phase B), 35 to 40 min (100% mobile phase B), and 40 to 60 min (20% mobile phase B). Samples were analyzed in duplicate, and results expressed as g amino acid per 100 g total amino acids.

### Fatty acid profiles

The triglyceride profile was determined by HPLC. The analysis was carried out using a HPLC (Infinity Series 1260, Aligent Technologies, Germany) fitted with an auto sampler and a Refractive Index Detector. The Hypersil ODS C-18 column at ambient temperature was used with mobile phase of Acetonitrile: Acetone (37.5:62.5) at flow rate of 1.5. The elution program was isocratic. The purpose of this analysis was to characterize the fatty acid composition. Using mole ratio, the concentration of each component fatty acid was calculated for each triglyceride [36]. All samples were analyzed in duplicate.

### Mineral analysis

The mineral analysis of iron, calcium, magnesium, zinc, and copper was conducted with atomic absorption spectroscopy [37]. The samples were dry-ashed in a muffle furnace at 550 °C for 6 h. The minerals were extracted from ash with 20 mL of 2.50% HCl and heated in a steam bath to reduce the volume to 8.0 mL, which was transferred quantitatively to a 50 mL volumetric flask and diluted to volume using deionized water. The extracts were stored in dry, clean plastic sample bottles, and the mineral concentrations were determined using an atomic absorption spectrophotometer. Potassium was determined with the flame photometric method [38] using a low temperature direct reading single channel emission flame photometer. Significant differences between the mineral contents of the three forms of *akokono* were analyzed using one-way ANOVAs.

### Vitamin determination

#### Vitamin a

Vitamin A was released from the sample by alkaline hydrolysis using ethanolic potassium hydroxide solution and extracted three times with hexane:ethylacetate (85:15 volume/volume %). The determination was carried out by normal-phase chromatography (NP-HPLC) with ultraviolet/diode array detector (DAD) at 325 nm (EN 12823–1:2014) [39].

#### Vitamin B1 (thiamine)

Thiamine was extracted from the sample in an autoclave using acid hydrolysis and quantified by reverse phase chromatography (RP-HPLC) coupled with a fluorescence detector (Ex.: 368 nm, Em.: 440 nm) after post-column oxidation to thiochrome (EN 14122:2006 with modification) [40].

#### Vitamin B2 (riboflavin)

Riboflavin was extracted from the sample in an autoclave using acid hydrolysis and quantified by RP-HPLC coupled with a fluorescence detector (Ex.: 468 nm, Em 520 nm) (EN 14152:2006 with modification) [41].

#### Vitamin B3 (niacin)

Niacin was calculated as the sum of nicotinic acid and nicotinamide. The two compounds were extracted from the sample in a mild hydrochloric solution at 100 °C, the pH of the extract was adjusted to pH 4.5 with sodium acetate followed by a filtration.

#### Vitamin B6

To determine vitamin B6, the European Union reference method for the determination of vitamin B6 in foodstuff (EN 14164–2008) was used with modification [42]. Briefly, the sample was hydrolysed in an autoclave followed by enzymatic dephosphorylation, reacting with glyoxylic acid in the presence of Fe^2+^ as a catalyst to transform pyridoxamine into pyridoxal, which is then reduced to pyridoxine by the action of sodium borohydride in alkaline medium. Total pyridoxine was quantified by RP-HPLC with a fluorescence detector (Ex: 290 nm, Em: 395 nm).

#### Folate

Folate was extracted from the sample in an autoclave using a buffer solution, followed by an enzymatic digestion with human plasma and pancreas V, and finally by a second autoclave treatment. After dilution with basal medium containing all required growth nutrients, except folic acid, the growth response of *Lactobacillus rhamnosus* (ATCC 7469) to extracted folate was measured turbidimetrically and compared to calibration solutions with known concentrations [43].

#### Vitamin B12

Vitamin B12 was extracted from the sample in an autoclave using a buffered solution. After dilution with basal medium (containing all required growth nutrients except cobalamin) the growth response of *Lactobacillus leichmanii* (ATCC 7830) to extracted cobalamin was measured turbidimetrically. This was then compared to calibration solutions with known concentrations [44].

#### Vitamim D3

The samples were saponified in alcoholic potassium hydroxide solution and extracted with hexane: ethylacetate (85:15 v/v%). The extract was concentrated and cleaned up by solid phase extraction (EN 12821:2009) [45]. The amount of vitamin D3 was determined by RP-HPLC with DAD (265 nm).

#### Vitamin E

Vitamin E was released from the sample by alkaline hydrolysis using ethanolic potassium hydroxide solution and extracted three times with hexane:ethylacetate (85:15 v/v%). Quantification was carried out by NP-HPLC with a fluorescence detector (Ex/EM 290 nm/327 nm) (EN 12822:2014) [46].

Significant differences between the vitamin contents of the three forms of *akokono* were analyzed using one-way ANOVAs.

### Ethics approval and consent to participate

This study was deemed not human subjects research by the PATH Research Determination Committee.

## Results

### Macronutrients, minerals, and vitamins

Table 1 presents the *akokono* nutrient content analysis from 100 g wet matter samples. The macronutrient profile of all forms of *akokono* was slightly more than half (53 to 56%) fat, one-third (32 to 33%) protein, and approximately one tenth (9 to 13%) carbohydrate. The fiber content ranged from 4 to 10%. The *akokono*-groundnut paste product had a slightly lower fat concentration than the unmixed forms of *akokono*, and similar protein and higher carbohydrate content.

**Table 1:** Proximate, mineral, and vitamin profiles of three forms of *akokono*^ǂ^

	Raw	Roasted	*Akokono*-Groundnut Paste^√^
Proximate^1^			
	g/100 g	g/100 g	g/100 g
Ash (%)	3.93 ± 0.13^a^	2.65 ± 0.09^b^	2.67 ± 0.19^b^
Fat (%)	52.72 ± 1.86^a^	53.04 ± 1.87^a^	46.34 ± 2.31^b^
Protein (%)	32.83 ± 1.16^a^	31.58 ± 1.11^a^	34.15 ± 1.71^b^
Carbohydrate (%)	10.52 ± 0.37^a^	12.73 ± 0.45^b^	16.84 ± 0.78^c^
Fiber (%)	9.61 ± 0.34^a^	4.52 ± 0.16^b^	3.94 ± 0.21^c^
Minerals^1^			
	mg/100 g	mg/100 g	mg/100 g
Iron^2^	0.85 ± 0.17^a^	1.30 ± 0.26^b^	1.50 ± 0.30^c^
Potassium	16.90 ± 0.85^a^	13.90 ± 0.70^b^	240.20 ± 12.01^c^
Calcium	4.60 ± 0.23^a^	–	42.90 ± 2.15^b^
Magnesium	25.80 ± 1.29^a^	23.30 ± 1.17^a^	126.50 ± 6.33^b^
Zinc	3.60 ± 0.18^a^	–	3.50 ± 0.18^a^
Copper	0.90 ± 0.05^a^	0.30 ± 0.02^b^	0.70 ± 0.04^c^
Vitamins^2^			
	mg/100 g	mg/100 g	mg/100 g
Vitamin A	< 0.021 (LOQ)	< 0.021 (LOQ)	< 0.021 (LOQ)
Thiamine	0.12 ± 0.02^a^	0.22 ± 0.04^b^	0.12 ± 0.02^a^
Niacin	4.79 ± 0.67^a^	4.02 ± 0.56^a^	7.88 ± 1.10^b^
Riboflavin	0.60 ± 0.10^a^	0.81 ± 0.13^a^	0.32 ± 0.05^b^
Vitamin B6	0.18 ± 0.03^a^	0.27 ± 0.04^b^	0.21 ± 0.03^b^
Folate	0.20 ± 0.06^a^	0.21 ± 0.06^a^	0.10 ± 0.29^a^
Vitamin B12	0.02 ± 0.00^a^	0.02 ± 0.01^a^	0.00 ± 0.00^b^
Vitamin D3	< 0.00025 (LOQ)	< 0.00025 (LOQ)	< 0.00025 (LOQ)
Vitamin E	11.20 ± 1.70^a^	18.60 ± 2.80^a^	68.70 ± 10.40^b^

^1^Tests were conducted by Microbiology Lab of the Department of Biochemistry and Biotechnology at KNUST on dry matter *akokono.* Results represent means of duplicate analysis

^2^Tests were conducted by Eurofins Lab on original matter *akokono*. Results represent means of triplicate analysis

^ǂ^Results are reported as mean ± sd. A one-way ANOVA was used to determine significant differences between the samples. Samples with different letter superscripts (a, b, c) in the same row are significantly different (*p*<0.05). The larval lifecycle stage was similar between all akokono tested.

*LOQ*: Limit of quantitation

^√^*Akokono*-groundnut paste was made with roasted *akokono*

In unmixed forms, *akokono* is richest in magnesium and potassium, followed by calcium, zinc, iron, and copper. When roasted *akokono* was combined with groundnut paste (and canola oil), the content of all analyzed minerals increased.

Of the vitamins present in *akokono*, the highest concentrations were observed for vitamin E (α-tocopherol) and niacin. There were small but significant amounts of thiamine, riboflavin, vitamin B6, folate, and vitamin B12 in all *akokono* samples; vitamin A and vitamin D3 were not present. When roasted *akokono* was combined with the groundnut paste and canola oil, vitamin E (α-tocopherol) and niacin concentrations increased, and thiamine, riboflavin, folate, vitamin B6 and vitamin B12 decreased. Roasted *akokono* alone had significantly greater concentrations of thiamine, riboflavin, and vitamin B-12 than when it was combined with groundnut paste and canola oil into a paste.

Table 2 provides a closer examination of the macronutrient and micronutrient contribution of one serving size of *akokono*-groundnut paste to the child diet. One serving size of two tablespoons (approximately 32 g) of *akokono*-groundnut paste contains enough protein to satisfy 99% of an infant’s (6 to 12 months) recommended dietary allowance (RDA) and 84% of a child’s (1 to 3 years) RDA. It also provides half (49%) of an infant’s adequate intake (AI) recommendation for fat (AI for children 1 to 3 years not determined). However, the same serving size only provides 7 and 5% of infant and child carbohydrate RDAs, respectively, and 7% of a child’s AI level for fiber (AI for infants not determined). It is a rich source of certain minerals (infant 6 to 12 months, children 1 to 3 years): copper (102% AI, 66% RDA), magnesium (54% AI, 51% RDA), zinc (37% RDA, 37% RDA), potassium (11% AI, 3% AI), iron (4% RDA, 7% RDA), and calcium (5% AI, 2% RDA). The paste also provides B-vitamins (infants 6 to 12 months, children 1 to 3 years): thiamine (13% AI, 8% RDA), niacin (63% AI, 42% RDA), riboflavin (26% AI, 20% RDA), vitamin B6 (22% AI, 13% RDA), folate (40% AI, 21% RDA), and vitamin E (a-tocopherol) (440% AI, 366% RDA).

**Table 2:** The nutritional content of one serving** *akokono*-groundnut paste in comparison to the recommended dietary allowance (RDA) or adequate intake (AI) for children 6–12 months of age and 1–3 years of age

	RDA or AI* standard		*Akokono*-groundnut Paste		
	6–12 months	1–3 years	32 g serving	%RDA or %AI	
				6–12 months	1–3 years
Macronutrients					
Fat (g)	30*	ND	14.83	49%	ND
Protein (g)	11	13	10.93	99%	84%
Carbohydrate (g)	95*	130	5.39	6%	4%
Minerals					
Iron (mg)	11	7	0.48	4%	7%
Potassium (g)	0.7*	3*	0.08	11%	3%
Calcium (mg)	260*	700	13.73	5%	2%
Magnesium (mg)	75*	80	40.48	54%	51%
Zinc (mg)	3	3	1.12	37%	37%
Copper (mg)	0.22*	0.34	0.22	102%	66%
Vitamins					
Vitamin A (ug)	500*	300	0.00	0%	0%
Thiamine (mg)	0.30*	0.50	0.04	13%	8%
Niacin (mg)	4*	6	2.52	63%	42%
Riboflavin (mg)	0.4*	0.5	0.10	26%	20%
Vitamin B6	0.3*	0.5	0.07	22%	13%
Folate (ug)	80*	150	32.00	40%	21%
Vitamin B12 (ug)	0.5*	0.9	0.00	0%	0%
Vitamin D3 (ug)	10	15	0.00	0%	0%
Vitamin E	5*	6	21.98	440%	366%
Fatty acids					
Linoleic acid	4.6*	7*	7.58	165%	108%

*Adequate intake (AI) values

**One serving = 2 tbsp, or approximately 32 g of *akokono*-groundnut paste

*ND*: Not determined

### Amino acids

Table 3 lists the concentrations of amino acids within two unmixed forms of *akokono* and the mixed *akokono*-groundnut paste in comparison to the concentration thresholds listed by the Institute of Medicine (IOM) for ASFs [47]. Concentrations of methionine and cysteine (sulfur amino acids) are examined together since methionine is often the most limiting amino acid in cereal grain-based diets, and cysteine is able to substitute for a portion of methionine’s requirement. Phenylalanine and tyrosine (aromatic amino acids) are also examined together. While both are indispensable to human functions, phenylalanine must be consumed in the diet, but tyrosine can be generated from the hydroxylation of phenylalanine [32]. Roasted *akokono* meets or exceeds the IOM’s concentration threshold for ASFs for four of the nine essential amino acids (histidine, isoleucine, phenylalanine + tyrosine, and valine), but concentrations of leucine, lysine, methionine + cysteine, threonine, and tryptophan fell short of the IOM threshold level. When *akokono* was combined with groundnut paste and canola oil, the concentrations of leucine, methionine + cysteine, threonine, and tryptophan increased to within 10% of the IOM threshold level; however, lysine content remained low. The concentrations of histidine, isoleucine, lysine, and phenylalanine + tyrosine were greater in unmixed *akokono* than the paste product. *Akokono* is also a source of seven non-essential amino acids, including alanine, arginine, aspartate, glutamate, glycine, proline, and serine (data not presented herein).

**Table 3:** Amino acid profile of three forms of *akokono*^a^ (mg/g protein), compared to the concentration of amino acids required to meet the IOM ASF definition

Essential amino acids	Raw	Roasted	*Akokono*-groundnut paste^√^	Concentration of essential amino acids required to meet IOM ASF definition^b^
	mg/g protein			
Histidine	52	50	32	18
Isoleucine	50	57	41	25
Leucine	40	21	51	55
Lysine	40	39	36*	51
Methionine + Cysteine	28	22	24	25
Phenylalanine + Tyrosine	115	97	91	47
Threonine	32	22	25	27
Tryptophan	–	5	9	7
Valine	35	36	40	32
Non-essential amino acids				
Alanine	108	140	69	–
Arginine	–	44	92	–
Aspartic acid (Aspartate)	47	46	94	–
Glutamic acid (Glutamate)	121	138	170	–
Glycine	37	31	48	–
Proline	91	132	70	–
Serine	108	140	69	–

^a^Values represent means of duplicate analysis

^b^Institute of Medicine (IOM). (2005). Dietary reference intakes for energy, carbohydrate, fiber, fat, fatty acids, cholesterol, protein, and amino acids. Institute of Medicine, The National Academies Press. Washington, D.C.

*Concentration of lysine in *akokono* -groundnut paste is more than 10% below IOM ASF definition

*ASF*: Animal-source food

*IOM* Institute of Medicine

^√^*Akokono*-groundnut paste was made with roasted akokono

### Fatty acids

Table 4 lists the fatty acid compositions of three different forms of *akokono.* The profiles of raw and roasted *akokono* respectively showed concentrations of 6.54 and 14.29 g/100 g linoleic acid (*n*-6 polyunsaturated fatty acid), 14.47 and 7.37 g/100 g oleic acid, 19.95 and 14.97 g/100 g myristic acid, and 19.52 and 13.87 g/100 g palmitic acid. When combined with the groundnut paste and canola oil, fatty acid concentrations (g/100 g *akokono*) increased compared to roasted *akokono* for linoleic (omega-6) acid (23.69), stearic acid (1.67), and oleic acid (11.16), while decreasing for myristic and palmitic acids (1.51 and 5.96, respectively). The fatty acid concentration of linoleic acid exceeds the AI levels for infants (165% AI) and children (108% AI). AI levels for oleic acid, myristic acid, and palmitic acid have not been set.

**Table 4:** Fatty acid compositions of three forms of *akokono*^a^

Fatty acids^b^	Raw		Roasted		*Akokono*-groundnut paste^√^	
	g/100 g	g/32 g	g/100 g	g/32 g	g/100 g	g/32 g
Linoleic acid	6.54	2.09	14.29	4.57	23.69	7.58
Oleic acid	14.47	4.63	7.37	2.36	11.16	3.57
Myristic acid	19.95	6.38	14.97	4.79	1.51	0.48
Palmitic acid	9.52	3.05	13.87	4.44	5.96	1.91
Stearic acid	0	0	0	0	1.67	0.53

^a^Values represent means of duplicate analysis

^b^Concentrations given in g fatty acid/g sample

^√^*Akokono*-groundnut paste was prepared with roasted *akokono*

### Feed experiment

Table 5 shows the results of the *akokono* feed experiments. *Akokono* fed on pito mash had > 10% increased concentrations of carbohydrate, potassium, calcium, sodium, and zinc concentrations in comparison to those fed on palm pith. The protein and iron content remained consistent between the two groups, but the fat content was lower among *akokono* fed on pito mash. The pito mash itself contained a higher concentration of fat, protein, and all measured elements.

**Table 5:** Proximate and mineral content of *akokono* and feed materials

	Palm pith	*Akokono* fed on palm pith	Pito mash	*Akokono* fed on pito mash
Proximates^a^				
	g/100 g	g/100 g	g/100 g	g/100 g
Ash (%)	8.08	3.23	3.46	3.18
Fat (%)	0.69	50.29	7.83	42.43
Protein (%)	24.08	31.83	27.82	32.39
Carbohydrate (%)	67.15	14.65	60.89	22.00
Elements^b^				
	mg/100 g	mg/100 g	mg/100 g	mg/100 g
Iron	0.48	1.69	0.69	1.75
Potassium	721.92	26.02	1562.70	65.95
Calcium	54.59	18.19	75.91	38.31
Sodium	77.52	19.99	92.28	42.48
Zinc	1.78	4.48	2.56	6.05

^a^Data represent mean of duplicate analysis reported on dry matter basis

^b^Data represent mean of duplicate analysis

## Discussion

This study presents the nutritional composition of palm weevil larvae (*akokono*), edible insects consumed in Ghana, and evaluates their adequacy as a complementary food, both alone and in combination with local groundnuts. We conclude that *akokono* alone does not provide a complete amino acid profile, but it does offer a significant amount of essential nutrients and can feasibly be integrated into agriculture and nutrition intervention strategies to address upstream determinants of health and nutrition. Furthermore, there is potential to improve the nutrient content of this food by manipulating feed inputs; however, further research in this area is warranted.

### Nutrient content

Consistent with existing research, our analysis determined Ghanaian *akokono* to be a nutrient-rich, high fat, high protein food. Roasted *akokono* contains adequate amounts of four essential amino acids (histidine, isoleucine, phenylalanine + tyrosine, and valine) but inadequate amounts of five essential amino acids (e.g., leucine, lysine, methionine + cysteine, threonine, tryptophan). Combining *akokono* with groundnut paste and canola oil favorably affected both the amino acid and fatty acid composition of the food, increasing the ratio of essential to non-essential fatty acids and the ratio of unsaturated to saturated fatty acids (i.e., more linoleic and oleic acid, less myristic and palmitic acid), likely largely due to the composition of canola oil. In comparison to roasted *akokono*, the paste product had greater concentrations of all minerals analyzed but lower vitamin concentrations (except for vitamin E and niacin).

In contrast to studies examining the nutritional composition of the palm weevil larvae in other African countries, the greatest differences are seen in mineral content, which varies between studies, independent of geography. We hypothesize that the larval diet could be one explanation for the variation between protein, fat, and mineral content. Our feed experiment indicated that the *akokono* responded to different feed substrates with changes in macronutrient and micronutrient composition. However, multiple studies from Nigeria [26, 29, 30], where the palm weevil larva were fed only raphia palm pith, still observed significant variability in mineral content. Research by Reynolds et al. [48] in Uganda found vast heterogeneity in mineral content between 11 samples of pith from raphia palms, suggesting that nutrient variability among the same type of feed substrate, perhaps due to soil quality, could also contribute to differences in nutrient profiles between different insect samples. Variability may also be linked to the developmental stage of the larvae analyzed [17].

We found that one serving (approximately 32 g) of *akokono*-groundnut paste provided 6% of the infant (6 to 12 months) iron RDA and 9% of the child (1 to 3 years) iron RDA. However, *akokono* is a potential source of highly bioavailable iron. Heme-iron is present in insect cytochromes [49] and ferrous iron is carried by holoferritin molecules in the vacuolar system and hemolymph [50]. Both forms have greater bioavailability than the ferric iron most prevalent in non-heme source foods [51], and the absence of iron-binding plant origin compounds [52] could be advantageous for the absorption of iron from *akokono*. The properties of insect iron may explain results from previous studies, including a trial which found that caterpillar fortified cereal improved hemoglobin levels, ferritin levels, and anemia prevalence among 18-month-old infants, [53] and an in-vitro study that showed iron bioavailability of several insect species to be comparable with that of sirloin beef [54]. It is plausible that similar benefits could extend to *akokono* presupposing that their underlying biology is largely the same as that of other insects.

### Adequacy as a complementary food

In many low-resource settings, women and children are deficient in iron, zinc, and vitamin A, and often subsist on diets low in protein and essential fatty acids. The nutrient profile of *akokono* can augment the intakes of certain micronutrients and macronutrients that play important roles in development and immunity. At the same time, concentrations of these nutrients may be dependent on insect feed and rearing practices. Opportunities to optimize the nutrient content of this food is an area in need of further research.

Based on our findings, *akokono* does not meet all the requirements to be classified as an alternative ASF; however, it still holds promise as a component of the complementary diet to help meet nutritional requirements. Pairing *akokono*-groundnut paste with lysine-rich foods can provide a complete protein profile. For example, potatoes and soybeans are two locally produced foods that are high in lysine. Additionally, it is also significant that the *akokono*-groundnut paste product meets the GSA requirements for commercial peanut butter products and can be safely consumed without refrigeration for 14 days (data not included). This suggests that the *akokono*-groundnut paste could be consumed in settings that lack electricity, without any added risk relative to other commercial peanut butter products in Ghana.

### Policies on insects as food

In Ghana, *akokono* has historically been harvested from palm trees; however, this practice has fallen out of favor among younger generations [22]. Recent efforts to stimulate the market have initiated the reintroduction of edible insects into the modern food supply in Ghana by way of microfarming. Forest foods can provide healthy additions to household diets and household microfarming can generate additional income; thus, opportunities for incorporating forest foods into nutrition policy and health intervention strategies should be further explored [55].

However, policies and legislation on insects as food are currently lacking on both national and international levels. While some institutions and policies, such as Ghana’s FDA, do focus on food standards in Ghana, legislation is generally broad and does not offer guidelines specific to insects. Although unprocessed *akokono* is recognized as a safe, local, traditional food that has been consumed for generations, GSA certification is required for all processed *akokono*-based products. Building quality control mechanisms and risk mitigation strategies can help to inform the standards enforced and upheld by the Ghana FDA and the GSA to ensure food safety. There is a great need for unifying regulations and efforts focusing on the production and sale of insects for human consumption, both in Ghana and internationally—particularly given their low environmental impact and potential to improve human nutrition.

### Limitations

This study investigated the macronutrient and micronutrient content of a small sample of domestically microfarmed *akokono*. Future research, including studies with larger sample sizes and those testing *akokono* harvested from the wild, is needed to confirm our results. In addition, further research into different feed inputs and their associated impact on the insect’s nutrient profile will continue to support both optimal insect nutrient content as well as inform low-environmental impact production efforts. Further analyses comparing the nutrient profile of *akokono*-fortified groundnut paste to that of groundnut paste alone may also provide additional insights. Acceptability studies, such as the one conducted by Bauserman et al., [56] are also warranted to further investigate consumer perceptions of *akokono*-fortified commercial food products.

In addition to further characterizing the nutrient profiles of *akokono* and *akokono*-fortified foods, clinical research is also needed to determine the impacts of these foods on human health and nutrition outcomes. For example, Bauserman and colleagues [53] evaluated the efficacy of a caterpillar cereal on stunting and anemia in the Democratic Republic of the Congo.

Finally, it was a limitation of this study that we did not investigate the digestibility of protein from *akokono,* nor did we examine micronutrient bioavailability. Although information on insect protein quality is limited, several studies have calculated Protein Digestibility Corrected Amino Acid Scores for various insect species [57, 58]. As such, insect protein quality and digestibility is an important area for future inquiry, particularly given the role of protein quality in early growth [59].

## Conclusion

Although there is no one-size-fits-all solution to malnutrition, it is important that we continue to examine traditional practices—such as entomophagy—and their implications for human and planetary health. In this paper, we conclude that the promotion and uptake of *akokono* as a complementary food could address nutritional inadequacies in the Ghanaian diet. Further, our feed experiment demonstrates the potential for using recycled by-products as novel feed inputs to reduce the environmental impact of production and manipulate insect nutrient profiles. Implemented properly, nutrition and policy interventions involving *akokono* could also incorporate nutrition-sensitive strategies centered around women-led micro-farming to generate supplemental income while improving food security.

## Data Availability

All data generated or analyzed during this study are included in this published article [and its supplementary information files].

## Abbreviations

AI: Adequate Intake
ASF: Animal-source food
DAD: Diode array detector
FDA: Food and drug authority
GSA: Ghana standards authority
HPLC: High-performance liquid chromatography
IOM: Institute of medicine
KNUST: Kwame Nkrumah University of Science and Technology
LMIC: Low- and middle-income country
LOQ: Limit of quantitation
ND: Not determined
NP: Normal-phase
RDA: Recommended dietary allowance
RP: Reverse phase
